# Review of Design Considerations for Brain-on-a-Chip Models

**DOI:** 10.3390/mi12040441

**Published:** 2021-04-15

**Authors:** Tiffany Cameron, Tanya Bennet, Elyn M. Rowe, Mehwish Anwer, Cheryl L. Wellington, Karen C. Cheung

**Affiliations:** 1 School of Biomedical Engineering, University of British Columbia, Vancouver, BC V6T 1Z4, Canada; tcam1@ece.ubc.ca (T.C.); tanyabennet@ece.ubc.ca (T.B.); 2 Centre for Blood Research, University of British Columbia, Vancouver, BC V6T 1Z4, Canada; 3 Department of Pathology and Laboratory Medicine, University of British Columbia, Vancouver, BC V6T 1Z4, Canada; elyn.rowe@ubc.ca (E.M.R.); mehwish.anwer@ubc.ca (M.A.); cheryl.wellington@ubc.ca (C.L.W.); 4 Djavad Mowafaghian Centre for Brain Health, University of British Columbia, Vancouver, BC V6T 1Z4, Canada; 5 Department of Electrical & Computer Engineering, University of British Columbia, Vancouver, BC V6T 1Z4, Canada

**Keywords:** brain-on-a-chip, microfluidics, extracellular matrix, basement membrane, endothelial cells, astrocytes, pericytes, neurons

## Abstract

In recent years, the need for sophisticated human in vitro models for integrative biology has motivated the development of organ-on-a-chip platforms. Organ-on-a-chip devices are engineered to mimic the mechanical, biochemical and physiological properties of human organs; however, there are many important considerations when selecting or designing an appropriate device for investigating a specific scientific question. Building microfluidic Brain-on-a-Chip (BoC) models from the ground-up will allow for research questions to be answered more thoroughly in the brain research field, but the design of these devices requires several choices to be made throughout the design development phase. These considerations include the cell types, extracellular matrix (ECM) material(s), and perfusion/flow considerations. Choices made early in the design cycle will dictate the limitations of the device and influence the end-point results such as the permeability of the endothelial cell monolayer, and the expression of cell type-specific markers. To better understand why the engineering aspects of a microfluidic BoC need to be influenced by the desired biological environment, recent progress in microfluidic BoC technology is compared. This review focuses on perfusable blood–brain barrier (BBB) and neurovascular unit (NVU) models with discussions about the chip architecture, the ECM used, and how they relate to the in vivo human brain. With increased knowledge on how to make informed choices when selecting or designing BoC models, the scientific community will benefit from shorter development phases and platforms curated for their application.

## 1. Introduction

In recent years, progress toward understanding human brain physiology and disease mechanisms has been advanced using animal models, and in vitro studies. The most common animal models are rodents, which are either studied as a whole animal or through use of primary cells harvested for in vitro studies. Mouse models are particularly appealing due to their low cost and the repertoire of genetically engineered strains for studying disease [1]. While there are efforts to “humanize” mouse models to make them more relevant to study human disease, a major limitation is that rodents do not naturally develop diseases seen in humans, and thus they are unable to recapitulate the complex series of events leading to pathologies such as Alzheimer’s disease. The human and murine brain also differ considerably in the proportion of gray:white matter, regional organization and gene expression [2]. The species-based limitations that accompany animal models have led to the widespread use of human-based in vitro models for exploring disease mechanisms and therapeutic development.

Human brain tissues can be modeled in vitro using organoids, where human induced pluripotent stem cells (iPSCs) or embryonic cells are differentiated into neural cell types that mimic the brain physiology in a 3D structure [3]. Neural organoids have become common tools for researching brain development and disease, with a focus on either localized regions or the complex interactions that occur between brain regions [4]. Unfortunately, using organoids for late-stage disease modeling is limited by nutrient and oxygen diffusion into the 3D structure, and incorporating functional vasculature into organoids is an ongoing area of exploration [5]. These limitations have influenced in vitro models to move towards a more controlled microenvironment such as brain-on-chip (BoC) models, where brain cells can be patterned to resemble the brain architecture and nutrients can be circulated throughout a microfluidic channel to mimic vascularization.

Transitioning from a relatively simple 2D monolayer culture—supported by widely available liquid handling and imaging systems—to a 3D microfluidic BoC model is more labor-intensive and costly. However, 3D models are capable of recapitulating important aspects of physiology, including flow over endothelial cells, and the space for neuronal and astrocytic projections. Further, this development can be made with relatively common materials, as the ability to pattern complex structures using soft lithography enables microenvironments to be compatible with a flow system by incorporating channels and ports into elastomeric materials such as polydimethylsiloxane (PDMS) polymers [6].

**Figure 1:**
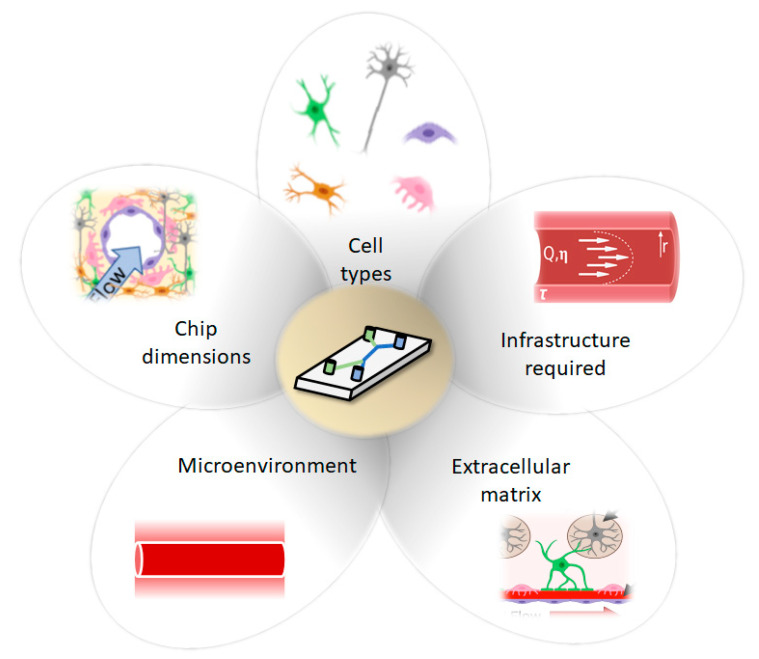
Considerations for development of Brain-on-a-Chip (BoC) models include cell types, chip dimension (2D, 2.5D, 3D), infrastructure required, ECM and microenvironment.

Recent advances in the development of microfluidic BoC devices and biological research have shed light on the importance of shear stress exerted on endothelial cells, substrate stiffness, and cell-to-cell contact for inducing the physiology that is observed in vivo. For example, BoC models have shown that shear stress exerted against brain microvascular endothelial cells (BMECs) plays a role in upregulating adherens and tight junction proteins [7], and modulating expression of blood–brain barrier (BBB) markers such as claudin-5 and glucose transporter 1 (GLUT-1) [8]. Several independent lines of evidence suggest that shear stress does not change BMEC morphology [9,10], but rather tightens the barrier; most often evaluated using trans-endothelial electrical resistance (TEER) and permeability assays [11]. Additionally, recent 2D in vitro studies have demonstrated that substrate stiffness plays a role in BMEC tight junction integrity as well as astrocyte and neuron morphology [12,13,14]. Transwell assays have also demonstrated the importance of cell-to-cell contact on BBB integrity, as several studies have shown that coculture of BMEC with astrocytes and pericytes can increase TEER and permeability measures [15]. Together, these findings suggest that contextual cues are critical when mimicking complex microenvironments such as the human brain. Throughout this review, we will outline recent advances in BoC models, as well as provide an overview of factors to consider when developing a BoC device (Figure 1).

## 2. BoC Development

**Figure 2:**
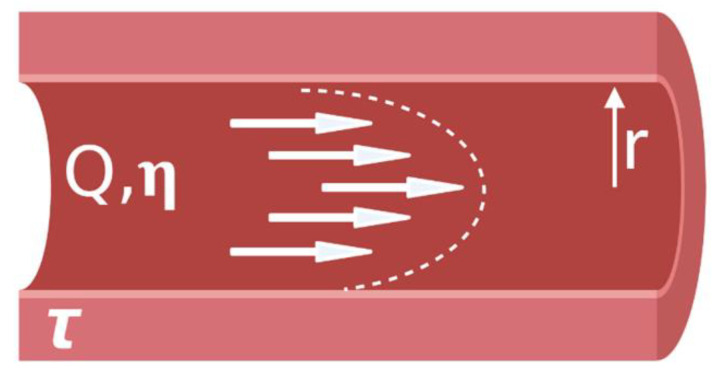
Parabolic shear stress profile in a blood vessel.

Advancing from a static 2D well-plate or Transwell experiment to a complex engineered platform that incorporates flow, requires careful initial consideration. Limitations of current models are discussed, so that new developments of BoC devices can aim to improve physiological relevance by selecting cell types, ECM, and a microenvironment that are similar to the brain in vivo. In this section, we will highlight the main considerations that are needed to build BoC devices, as well as highlight some of the current state-of-the-art BoC platforms and their limitations. To initiate development of a microfluidic BoC platform, the system requirements should be understood. The major initial considerations for BoC modeling include determining the region of interest within the brain and the corresponding vascular shear stress in that region. This will influence the BoC geometry and the pump specifications (syringe or peristaltic) needed to incorporate flow into the system. To decide on flow rate and BoC dimensions, the appropriate shear stress equation should be used. For instance, for a rigid, uniform, cylindrical vessel, the shear stress (τ) at the vessel wall can be derived from Poisseuille’s law to become:

τ = 4Q *η/πr^3^ (1)

where Q is the flow rate, η is the viscosity, and r is the radius of the vessel (Figure 2).

For example, to achieve a capillary-like shear stress of 1 dyn/cm^2^ [16] using a vessel with a radius of 100 μm, and standard cell media composition (Dulbecco’s Modified Eagle Medium (DMEM)) supplemented with 10% fetal bovine serum (FBS), which has a viscosity of ~0.93 mPa*s [17], then an approximate flow rate of 304 μL/hr would be required. However, if investigations lead to capillary vessel diameter changes, as seen with pericyte-mediated contractility, then adjusting the flow rate to obtain the desired shear stress may be needed [18]. To mimic blood flow in the brain, a continuous, unidirectional flow system is desirable to achieve physiological relevance. However, the concentration of soluble substances will be diluted as a function of flow rate and, at high flow rates, may fall below the detection limit of quantitative measures such as enzyme-linked immunosorbent assay (ELISA). Use of a peristaltic pump to continuously circulate fluid throughout the system may improve end-point analyses, as analytes would become concentrated in the circulating media.

During the prototyping stage, microfluidic chips are often fabricated using polydimethylsiloxane (PDMS). PDMS is a transparent, biocompatible, oxygen-permeable polymer that can be easily molded into high resolution geometries [6]. Creating a negative mold pattern is often performed using photolithography with a UV-sensitive material, patterning the silicon wafer with high resolution features in the micron range [19]. More recently, lower cost, 3D printed molds have been used to fabricate geometries in the range of hundreds of microns [20]. Alternative fabrication materials may be desirable, such as thermoplastic or polyester elastomers, if optical clarity or low adsorption is essential, although this will likely increase the cost per chip and affect the ease of fabrication [21]. Thermoset polymers such as SU-8 and thermoplastic polymers such as PMMA, polystyrene, and polytetrafluorethylene have also been used in microfluidic devices with improved solvent resistance, reduced small molecule adsorption, and improved rigidity compared to PDMS [22]. Teflon microfluidic chips, which will not adsorb small molecules, have been made using thermal compression [23], and PMMA chips have been made by thermal compression or laser micromachining. For research use, PDMS prototypes represent a reasonable compromise between the ease of production and low cost with performance of the device. The drawbacks of small molecule adsorption can be overcome with a coating on the PDMS or other postprocessing step. For commercial high volume manufacturing, the reliability that can be offered by other polymers such as polystyrene will become more important, and economy of scale justifies the investment in injection molding of polystyrene chips.

**Figure 3:**
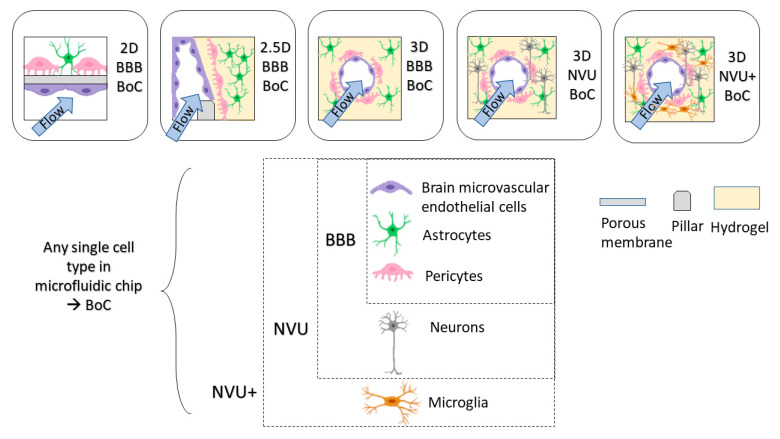
Dimensions of BoC devices. BoC devices can range from 2D, 2.5D, 3D with specific cell types to model BBB (EC, pericytes, astrocytes) or NVU (EC, pericytes, astrocytes, neurons).

Throughout this review, we will be classifying 2D, 2.5D and 3D BoCs based on their microfluidic chip architectures. We will term models that have ECs, astrocytes and pericytes as BBB BoCs and models that include ECs, astrocytes, pericytes and neurons as a neurovascular unit (NVU) BoCs (Figure 3). Depending on the cell types included in the model, consideration should be given to the extracellular matrix (ECM), which is a key component of the brain microenvironment that must be incorporated into BoC models to recapitulate physiological phenotypes. These considerations will be covered in subsequent sections, following an overview of the advances and limitations of existing BoC designs.

Planar cell layers (2D) are amenable to simple fabrication processes and are the easiest transition from a static 2D model. The design of a 2D microfluidic BoC typically includes two compartments separated by a permeable membrane permitting cell–cell interaction, where at least one compartment acts as a flow channel to mimic vascular blood flow [11,24]. Commonly used membrane materials include polycarbonate (PC), polyester (PET) and PDMS membranes. In previous 2D BoC microfluidic models, PET and PC membranes were cut out of Transwell inserts to use in microfluidic BoCs [25]. More recently, commercially available track-etched PET and PC A4 sheets have been used [26,27]. To bond porous membranes to PDMS to obtain a leak-free channel, spin-coating PDMS [28], aminosilanization [29] or custom bonding procedures [30] may need to be performed. Furthermore, porous membranes such as PDMS will need further treatment to achieve the hydrophilicity required for adherence of ECM coatings [31]. The pore size and thickness of the porous membrane should be selected based on the application, as contact of astrocytes and pericytes with the endothelial cell monolayer will influence BBB function [32], thus larger pore sizes (~3 μm) should be considered to enable contact and increase fidelity to the native BBB anatomy. Commercially available microfluidic chip options such as the Emulate platform use a PDMS chip with 7.0 μm pore size in the membrane separating the channels [33]. Another important consideration in a 2D model is the ECM coating used on the membrane to mimic the microenvironment of the human brain basement membrane. Recent studies have explored the effect of ECM composition on endothelial cell tight junction properties [34], as well as permeability in Transwell inserts [35]. However, there are limited investigations into ECM coatings appropriate for coculture and tricultures in 2D environments that incorporate flow. Further exploration into ECM coatings used for BBB models will strengthen the robustness of 2D BoC models and potentially provide reliable environments to establish in vivo-like cellular functions and gene expression.

In this review, the term 2.5D model is used to describe endothelial cells forming a flat 2D monolayer around a rectangular channel that contains a 3D matrix [36]. 2.5D models are often used to recapitulate the architecture of the brain parenchyma by using a parallel channel design containing a hydrogel in one channel and flow across endothelial cells in the other channel. This design uses pillars to create distinctions between channels, so that a hydrogel can be flowed into the channel and cured using thermal gelation, photocrosslinking or chemical crosslinking methods. A 2.5D design allows brain cells to migrate towards the endothelium through a hydrogel to provide direct cell-to-cell contact as an artificial membrane is not required [37]. Having a planar 2.5D model will also improve imaging, since the media supply is parallel to the cells, compared to underneath and on top of the cells in 2D BoC models, which increases the working distance from the microscope focal point and may require imaging through additional layers of PDMS. Adriani et al. used a 2.5D model to embed primary rat astrocytes and neurons in a collagen-I hydrogel using microscale trapezoidal PDMS structures that acted as phase-guides to create a hydrogel network along their flow channel [38]. The commercially available Mimetas Organoplate^®^ platform also enables astrocytes and pericytes to be embedded into a collagen-I hydrogel, and endothelial cells to be seeded adjacent to the gel, and bidirectional flow is achieved using a rocking plate [39]. Yoojin et al. developed a microfluidic chip with five parallel channels to study BBB dysfunction in Alzheimer’s disease [40]. The use of collagen-I as a hydrogel has been largely investigated based on its structural integrity when gelled at a high concentration (>4 mg/mL). However, collagen-I is not found in the brain microvascular ECM; therefore, there is need for a hydrogel that can maintain its form while in a gelled state. Lee et al. have used a fibrin-based 2.5D model that was able to support angiogenic behavior [41]. Moving away from collagen-I-based 2.5D models will enable a more physiologically relevant brain compartment, where further insights can be gained into the functionality of neurons, mural cells, and glia.

A 3D BoC consists of a 3D matrix completely surrounding a perfusable circular cross-section of the endothelial cell layer. Several methods can be used to develop BoC with a circular cross-section, including using a needle as a sacrificial mold within a hydrogel [37,42,43,44,45,46] and using gravity-driven pressure to displace the hydrogel, also known as viscous fingering [47]. Notably, there are recent 3D BoC models that contain immortalized or primary endothelial cells [42,43,45,46,47,48], pericytes [42,45,47] and astrocytes [42,43,46,47,48], and some progress is being made in including iPSC-derived cells in 3D BoC devices [44,49].

One of the major challenges with creating 3D BoCs is the ability to select a hydrogel that is sufficiently mechanically stable to withstand perfusion while also providing a physiologically relevant ECM for cell growth. As in 2.5D systems, collagen-I is also commonly used as an ECM that encapsulates either astrocytes or pericytes in a 3D BoC architecture [42,47,48,50]. A mixed matrix of collagen-I, Matrigel^®^ and hyaluronic acid that supports astrocyte growth in 3D and endothelial cell growth on the inner lumen has also been developed [43,46]. Studies on mechanically stable hydrogels other than collagen-I that can support brain cells are of high interest. For instance, human umbilical vein endothelial cells (HUVECs) and brain pericytes were successfully cultured in a fibrinogen matrix that could withstand perfusion for up to 7 days [45].

Notably, all of the previously mentioned 3D BoC models lack neurons. To identify viable options for hydrogel-based ECM for BoC devices that contain neurons, knowledge gained from other in vitro modeling fields should be incorporated into BoC models. For example, there have been advances in angiogenic brain models that use biocompatible hydrogels to culture combinations of iPSC-derived endothelial cells, astrocytes, pericytes, microglia, and neurons in 3D [51,52]. In addition, Arulmoli et al. have demonstrated mechanical compatibility and biocompatibility of a salmon fibrin/hyaluronic acid/laminin hydrogel that could support iPSC-derived neurons and was in the brain stiffness range [53]. Furthermore, O’Grady et al. developed a gelatin-based, N-cadherin hydrogel that supported significant outgrowth for cultured neurons compared to conventional biomaterials such as Matrigel^®^ and had the mechanical stability to form a lumen [54]. To streamline this research for use in BoC models, an effort should be made to define and report mechanical properties of hydrogels in development so that mechanical dependencies of brain cells can become more defined for future development.

## 3. Decision Workflow: Factors to Consider When Selecting a Model System

It can be a daunting task to select or design a BoC device to address a specific biological question. This section aims to guide the selection of an in vitro model best suited for a given biological question and application. The intention is to eliminate the need for significant trial and error and allow for a streamlined development or selection of a minimum viable model (MVM). An MVM can be defined as the required model components that are essential to answer a biological question. For example, if the goal is to examine the toxicity of a compound, a 2D monolayer high throughput screen could be an appropriate starting point. The MVM does not necessarily represent the optimal model, but rather constitutes the base model with the minimum level of complexity needed to answer the desired question(s). Additional model complexity can be added to gain further biological insight, although this often increases both cost and experimental variability [55]. Ideally, the device should mimic the in vivo microarchitecture and microenvironment as closely as possible while demonstrating predictability, reproducibility and robustness. A stepwise approach to increase throughput or enhance physiological relevance can avoid introducing multiple variables at once.

**Figure 4:**
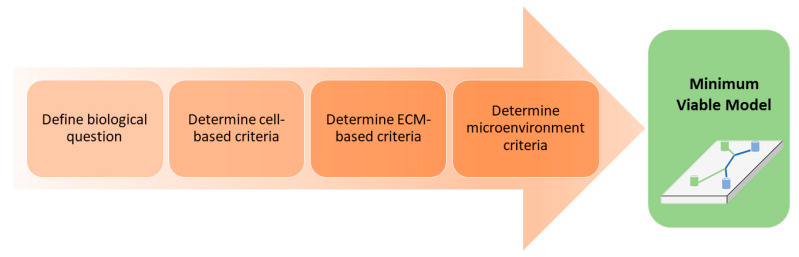
Steps for determining the minimum viable model (MVM).

To ensure confidence in the selection and/or design process, we aim to better inform the decision-making process by highlighting the benefits, constraints and limitations of various model components (Figure 4).

### 3.1. Biological Question

The first step in selecting a BoC system is to consider the goal of the experiment and the limitations of different BoC systems (Box 1). For example, BoC platforms of various designs and complexities have been used for studies on transport across the blood–brain barrier [39,56], cancer cell and immune cell invasion [57], drug screening [11] and disease modeling [58,59]. Each application may require a different MVM given the end-points, timeline and throughput required. Generally, throughput and complexity are inversely related, with simpler systems selected for high throughput experiments (i.e., drug screening) and complex systems containing multiple cell types and physiological ECMs for lower throughput experiments that focus on understanding physiological/pathological processes. Most microfluidic-based BoC models are not designed for high throughput; however, models that fall into the 2.5D category or simply eliminate flow can be adjusted to meet throughput needs. For example, the Mimetas Organoplate^®^ platform simplifies flow in order to increase its throughput capacity [39].

Box 1 **Factors to consider when selecting a BoC model system.**

**Application**


What is the purpose of the experiment?
◦Examples:
▪Investigate biological processes such as tissue repair or cell migration.▪Investigate cell response to drugs.▪Disease modeling.



B.
**Endpoints**


What endpoints are needed for the application?
◦Examples:
▪Localization of proteins (Immunocytochemistry).▪Permeability.


What kind of assays exist for this endpoint?
◦Example:
▪Fluorescein isothiocyanate (FITC)-dextran permeability assay.▪Monocyte adhesion.


What kind of samples are required for the assay?
◦Examples:
▪Monoculture.▪BBB (EC, pericytes, astrocytes).▪NVU (EC, pericytes, astrocytes, neurons).


Will there be real time measurements or an endpoint measurement?

C.
**Throughput**


How many conditions will be tested?How many samples are needed for the analysis?

D.
**Experimental timeline**


How long will the experiments take to get a result?How long does it take to establish the cell culture?

Important Note(s):

- Even though PDMS is a common material found in BoC platforms, its drawbacks include absorption of some proteins and small molecules [60].
- Real time analysis can be carried out with integrated electrochemical sensors [61] or fluorescence microscopy, among other methods. If this analysis is carried out by visualization (i.e., real time visualization of barrier function), it is important to select or design a device with desirable optical properties (i.e., optical transparency, thickness within the working distance of the microscope that will be used for visualization).
- If the biological question requires dissection of the contributions of individual cell types, independent access/channels for each cell type are required or different chips that represent various portions of transport can be utilized [56].

### 3.2. Cell-Based Criteria

A major challenge in designing a relevant model for a specific application is choosing the appropriate cells to form its basis. The first step is determining whether the cells and their specific origins are required or desired. Brain microvascular endothelial cell (BMEC) monocultures are suitable for testing toxicity [62] and proliferation [63], but this model often lacks the cell-to-cell signaling that is required for BMECs to achieve their full barrier potential [64,65]. Often, when a research question is associated with the brain microvasculature, a BBB model is used to answer mechanistic [52,66] or transport [67] related questions. Here, we define the functional unit of a BBB to be a triculture of BMECs with pericytes and astrocytes, since these supporting cells are critical in maintaining the highly selective barrier in vivo, through many mechanisms (reviewed in [68]). If neuronal signaling or crosstalk with the vasculature is a focus of the study, then a neurovascular unit (NVU) consisting of BMEC, pericytes, astrocytes, and neurons should be used. Differentiation of specific cell types from induced pluripotent stem cells (iPSCs) has been a major advancement in modeling the human cerebrovasculature in vitro, but several limitations still exist with these new techniques. In this section, we highlight the main contributions of each cell type to the defined units and review the advantages and limitations that govern cell selection options (i.e., immortalized, primary, iPSC-derived).

Brain endothelial cells (BECs/BMECs) line the inner walls of the cerebrovasculature and establish the highly selective barrier for entry into the brain. Single-cell transcriptomics has demonstrated that these cells have a gradual phenotypic change along the transition from artery to capillary to vein [69], but most BoC models aim to recapitulate the capillary, so brain microvascular endothelial cells (BMECs) will be the focus of this section. When selecting BMECs for use in a BoC, they should express key markers seen in vivo, including endothelial cell-specific markers (PECAM-1, VE-cadherin), tight junction markers (claudin-5, occludin, ZO-1), and key transporters (GLUT1, P-glycoprotein, LRP1, MFSD2A, BCRP), in addition to their ability to form a confluent monolayer with a tight barrier. Measuring barrier function is often performed on a Transwell insert, where transepithelial/endothelial electrical resistance (TEER) measurements can be obtained as a global measure of barrier integrity. Animal studies suggest that a physiological TEER value of the brain microvasculature is between 1500 and 8000 Ω·cm^2^ [70,71], compared to peripheral capillary vessels, which have TEERs of 2–20 Ω·cm^2^ [72,73].

As for sourcing BMECs, it is well-established that human immortalized and primary BMECs cannot achieve physiological TEER or permeability values in vitro, even when tricultured with pericytes and astrocytes (reviewed in [74]). Despite this limitation, immortalized cell lines (HCMEC/D3, TY10, BB10, HMEC-1) and primary human brain microvascular endothelial cells (HBMECs) continue to be used to identify changes in barrier integrity by measuring relative values before/after a treatment or disruption [40,75,76,77]. Immortalized cell lines are an attractive option due to their low cost, ease of use, and their ability to be passaged multiple times while retaining BBB transporter expression [78,79]. However, their monolayer permeability is much higher than physiological levels, with an average TEER of <40 Ω·cm^2^ [74], indicating that there is likely paracellular transport due to immature tight junction formation. Recent effort has been made to optimize immortalized BMECs by altering their culture conditions to improve barrier functions. For instance, Hinkel et al. have cultured HCMEC/D3s in static and dynamic conditions, adjusting the cell culture media, adding supplements to the media, adding ECM coatings to cultureware, and coculturing with astrocytes, but none of these conditions improved their TEER [80]. This is in agreement with earlier work demonstrating that coculture of BMEC immortalized cell lines with astrocytes or pericytes did not improve their TEER [40,75,76,77]. Another study identified transcriptional differences in HCMEC/D3 compared to primary human BMEC in genes that regulate the immune response, which seem to be directly related to the immortalization procedures used to create the cell line [81]. Taken together, these results suggest that immortalized human BMECs are not a suitable model for investigation of permeability or BBB transport and may not accurately depict a physiological inflammatory response.

Primary cells are another option as a source of human BMECs. The main advantages of primary HBMECs are that they are directly derived from human brain microvessels (often from temporal lobectomies), have not been altered by immortalization, and express the majority of the defining BEC markers in vitro [82]. However, the limited availability of human cerebral tissue makes cell sourcing challenging and introduces between-donor variabilities. Further, primary HBMECs also do not achieve physiological TEER, even under various optimized culture conditions [12,34,74]. Another major concern for primary cells is their dedifferentiation in vitro, which has been well-documented by many groups [81,83]. Human umbilical vein endothelial cells (HUVECs) are another common course of human endothelial cells for BBB/NVU models, which may acquire some characteristics of BMEC when in co- or triculture [84]. However, to date these HUVECs have not been shown to form a tight barrier even in the presence of both astrocytes and pericytes [52,66], and behave differently than HBMEC in several contexts [85,86].

To achieve an endothelial barrier that recapitulates the in vivo context, the best option to date is iPSC-derived BMECs [26,44,87,88,89,90,91]. While TEER values within the physiological range has been achieved by some of these differentiation protocols, a recent paper by Lu et al. demonstrates that the resulting cells from all protocols lack some key characteristics of endothelial cells and appear to be more closely related to epithelial cells [92]. Yet, these cells remain the only BMECs in culture that have both a strong barrier and functional BBB transporters [93] and will likely continue to be the gold standard until a better protocol is validated. Maximum barrier maturity with these cell types has taken up to 11 days in culture following differentiation [94], which will influence the experimental timeline and design considerations for the BoC model.

Astrocytes—named after their star-shaped morphologies—are the most abundant cell type in the brain. They play many critical functional roles, including reinforcement of the BBB [95,96], regulating cerebral blood flow [97,98], responding to inflammation [42,99,100], maintaining molecular homeostasis through regulating ion and pH balance [98,101], and supporting neurons by facilitating synaptic stability and plasticity [102,103]. Astrocytes extend their endfeet to contact and ensheath cerebral vessels [104] and have classically been considered as essential components in the physical barrier of the BBB. However, a recent mouse study that removed endfeet from cerebral vessels using a laser found that the vessels did not become more permeable [105], suggesting that it is their effect over time on endothelial cells—likely through secreted factors that could be soluble or components of ECM [106,107]—that reinforce the BBB. In vitro, astrocyte contact or noncontact coculture with BMECs from various origins has been shown to increase tight and adherens junction gene expressions and global permeability measures [94,108,109,110,111,112] further illustrate that astrocyte crosstalk with endothelial cells is critical for BBB physiology. Given their established roles in facilitating neurovascular coupling [97,98,113], astrocytes are key components in NVU models. There are many nuances in astrocyte classification depending on function and brain region, which is beyond the scope of this review. Several excellent reviews have recently been published on astrocytes in physiological and pathological contexts [68,98,114,115,116,117].

To date, most in vitro work with astrocytes has been carried out using mixed glial cells harvested from early postnatal rodent pups, which become enriched to approximately 95% in astrocytes during culture but do not achieve purity. While many BBB/NVU models have used this approach [118,119], recent work has highlighted both transcriptional and functional differences between human and murine astrocytes [120], which limit the capacity of rodent-based models to suitably mimic human physiology and disease. Therefore, here we will focus on cells of human origin. To validate the astrocyte identity, the most common marker is glial fibrillary acidic protein (GFAP), which is the major intermediate filament protein in astrocytes that is upregulated when they are in a reactive state. However, GFAP is not expressed in all mature human astrocytes [121,122]; therefore, a panel of additional astrocyte markers including S100-beta and NDRG2 [120,123] is recommended to confirm astrocyte identity prior to use. Sources of human astrocytes include commercial immortalized (astrocytoma) cell lines [124,125,126] and primary cells [127,128,129], as well as a growing collection of published protocols to generate iPSC-derived astrocytes, [130,131,132,133] as reviewed in [134,135,136]. Several new differentiation protocols have been published within the last year, including Gatto et al. who showed that direct differentiation of astrocytes from fibroblasts retained the age-related transcriptional differences of their donors [137].

The main limitations of culturing astrocytes from any source are purity and reactivity, as discussed in a review by Guttenplan et al. [123]. While obtaining pure isolates of primary human astrocytes is now possible due to advances in cell isolation methods [120,138], these methods are expensive for commercially sourced cells. Differentiation protocols have also advanced to produce pure astrocyte cultures [138], but these are labor-intensive and can be costly. If purity is essential and cost is a limitation, one option is to use an immortalized line, albeit with lower physiological relevance. Another option is to introduce neural precursor cells into the model, whereby the cells will differentiate within the model itself [139]. In this case, the result would be a mixed population of cells, which is suitable for a NVU model, but may limit the ability to identify astrocyte-specific effects. Astrocyte reactivity is a limitation across the map in vitro, as culture with serum or on stiff substrates will induce reactivity [123,140,141]. To date, the option to best mimic astrocytes in their quiescent state is to culture them in a hydrogel in either a 2.5D or 3D model [141,142,143].

Pericytes are mural cells embedded in the basement membrane of microvasculature. The cerebrovasculature has significantly higher pericyte coverage than peripheral vessels [64,144], which underscores their functional importance in the brain. Over the last decade, pericytes have gained considerable attention for their critical role in maintaining BBB integrity [144,145], as it has also been shown that increases in BBB permeability with aging can be traced to pericyte loss [146]. In vivo, pericytes guide astrocytic end feet and mediate their polarization [147], as well as induce specific transporter (Mfsd2a) expression in BMECs to promote a selective barrier phenotype [148]. The reinforcing effect of pericytes on in vitro BBB integrity has also been observed by many groups with various cell sources [37,88,149,150]. The interaction between pericytes and BMEC is likely very complex, as Yamazaki et al. recently showed that pericyte genotypes can influence BMEC barrier integrity by altering the secreted ECM [151]. These lines of evidence showcase the critical interaction among pericytes, astrocytes, and endothelial cells in the BBB. In addition to their roles in preserving a functional BBB, pericytes regulate cerebral blood flow and capillary diameter [18,152,153] and are involved in the immune response [42,154], among other functions (reviewed in [155]). Importantly, the contribution of pericyte dysfunction to the neuropathological features of stroke and Alzheimer’s disease is being increasingly recognized [156]. Therefore, a BoC model without pericytes will limit insights into physiological and pathological functionality of the BBB, and effectively the NVU.

A main issue in the use of pericytes in BBB/NVU models is their heterogeneous natures [157] and the lack of consensus on defining markers of this cell type for use in culture. Vanlandewijck et al. published an elegant single-cell transcriptomics paper which defined the zonation differences in mural cells along the murine cerebrovasculature and compared brain-derived pericytes to those from the lung [69]. They found many differences between pericytes from the brain and lung, reinforcing the idea of organotypicity in cells [158]. To confirm brain pericyte identity in vitro, no single marker is sufficient due to the overlap of markers with other mural cell types, but rather a combination of PDGFRβ, CD13, CD146, and NG2 expression is recommended [159]. As for cell source options, there are immortalized, primary, and iPSC-derived options available. Immortalized human brain pericytes are available from several vendors and have been used in many studies [39,149], but a new line (HBPC/ci37) recently developed and characterized by Umehara et al. [160] is another option. Primary human pericytes are also commercially available and are widely used [42,161,162]. As for iPSC-derived pericytes, the unclear definition of pericytes has resulted in all recent differentiation protocols being limited to the titles of “pericyte-like” [163,164] or “mural cells” [52,66], but these cells have been successfully incorporated into BoC models [37].

Neurons are often considered as the functional unit of the brain, and many have argued that without neurons, any given model does not truly represent the brain. Here, we make the distinction between modeling the BBB and the NVU, whereby the neurons are not essential for BBB formation, but are indispensable in modeling the NVU. Neurovascular coupling is the relationship between neurons and other cells comprising the BBB, which ensures that the highly metabolically active neurons are supported by sufficient oxygen and nutrients from the bloodstream. Neurons communicate their metabolic demands directly to endothelial cells or indirectly through astrocytes and pericytes to induce vasodilation that regionally increases blood flow where required [165]. Other cell types and the ECM of the NVU can also directly influence neuronal signaling [166]. This complex interplay is disrupted in many diseases [68], which increases the importance of near-physiological modeling of the human NVU. In addition to their communication with other cell types of the NVU, a key feature of neurons is their communication with each other. Neurons communicate via synaptic transmission, and they are classified into different types based on the neurotransmitters they secrete (e.g., dopaminergic neurons, glutamatergic neurons, etc.), as well as their location in the brain (e.g., cerebellar, cortical, hippocampal, etc.). Neuronal classification is beyond the scope of this review but has been discussed in several other reviews [167,168] and is an important factor to consider when designing an NVU model. Ideally, neurons should be derived from the brain region of interest and signal with the neurotransmitter(s) of interest, relevant to the biological question to be answered.

Similar to the other cell types discussed, significant differences between rodent and human neurons have been identified in recent years [169]. As for human-derived options, the neuroblastoma line SH-SY5Y is a relatively cheap and commonly used source of neurons in vitro, which retain the expression of many neuronal markers and can be further differentiated into more specific neuron classes (reviewed in [170]). These attributes have recently been leveraged to explore BoC model variations by Bastiaens et al. [171], highlighting their utility in model development rather than answering complex biological questions. Human fetal primary neural stem/progenitor cells, including the commonly used HIP-009 line, are also a popular choice used by several labs [56,169,172,173] and have been incorporated successfully into a BoC model [56]. There are also many emerging differentiation protocols for generating a variety of different types of neurons from iPSCs [174,175,176,177,178], which are beginning to be used in disease modeling [179,180] and toxicity testing [181].

Ultimately, all sources of neuronal cells will require some level of differentiation to generate well-defined cell types either before introduction into the model or differentiate while in the model. Differentiations from iPSC can take months and experimental timeline design considerations should account for the possibility of further differentiation within the model.

Microglia are the resident immune cells of the brain and are currently a main research focus in understanding underlying and potentially treating neurodegenerative diseases [182,183,184]. Activated microglia are seen as both a cause and consequence of BBB dysfunction in neurological disease [185], so their incorporation into BoC models is often desired to fully understand disease processes. In fact, a recent study in a mouse model has shown that microglia play a dual role in the context of systemic inflammation, first reinforcing the BBB at an acute phase, then disrupting it during chronic inflammation [186]. However, recapitulating adult human microglia in vitro to further study the relationship of microglia with the cerebrovasculature has been very challenging due to many technical limitations. A discussion of these issues is beyond the scope of this review, but if microglia incorporation into the BoC model is required, we refer the readers to several excellent reviews on this topic [187,188,189,190]. 

Overall, it is important to note that even after the required cells are incorporated into the device, each cell type should be validated for retention of in vivo-like phenotypes and behaviors, including marker expression and functional outcomes (e.g., endothelial cell transport, neuron signaling), as 3D cocultured environments are increasingly appreciated to alter cell gene expression and phenotypes compared to classical 2D monocultures. Therefore, the overall complexity of the model should only be increased once an acceptable level of validation is achieved. Transitioning from immortalized cell line-based proof-of-concept models to primary or iPSC-derived models will also require additional optimization steps, as these cells will behave differently than the robust immortalized lines. Optimization may relate to cell seeding densities, the basement membrane (BM) or ECM composition, adding physiological flow, or even considering microenvironmental criteria such as oxygenation and media composition. Furthermore, no single-cell type functions independently; barrier formation and neuronal function depend on multicellular interactions among cell types of the NVU [191,192]. Therefore, mimicking cell–cell interactions is just as vital as incorporating the cells into the model. Once the required cells and cell origins have been determined, the focus should turn to the interactions of interest. Interactions within a given cell type (homotypic) and between cell types (heterotypic) of the BBB or NVU can be replicated by leveraging microfabrication techniques to control tissue organization and structure. The 3D multicellular tissue architecture of the brain is best recapitulated using 2.5D and 3D models through the use of hydrogels, to maintain cells in a physiological orientation. If replication of cell stratification/organization is required, the MVM should contain independent cell access and segregation methods (i.e., using pillars to separate hydrogel and media channels) to ensure cells are seeded as they appear in vivo. In addition to replicating cell–cell interactions, many applications require modeling of cell–ECM interactions to interrogate the biology of the brain microenvironment or disease pathophysiology. When interested in investigating cell–ECM interactions (described in the following sections) it is vital that the MVM contain an appropriate ECM composed of natural biopolymers. Models become limited in their ability to replicate cell–ECM interactions in addition to other crucial ECM properties when synthetic materials (i.e., porous polyester membranes) are used as ECM substitutes.

### 3.3. Extracellular Matrix Criteria

**Figure 5:**
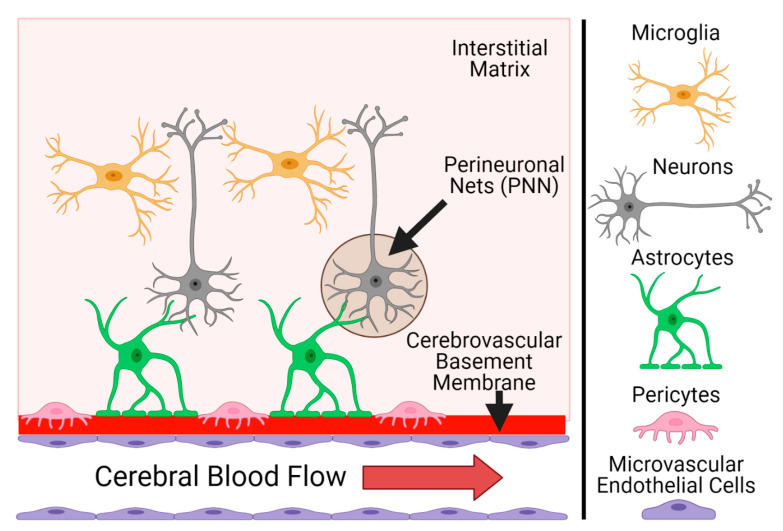
Brain ECM consists of a basement membrane, perineuronal nets and the interstitial matrix.

The extracellular matrix of the brain, initially known as the “ground substance”, constitutes about 20% of the total brain volume [193,194] and serves as a microenvironment constituted of glycans and proteins (hyaluronic acid, proteoglycans, linker proteins) secreted by neurons and glial cells. The ECM not only anchors the cellular components of the brain, but also facilitates fundamental CNS processes such as neuronal development and synaptic plasticity [195,196]. During development, the ECM serves as an enriched environment for survival and maintenance of neural stem cells and modulates their differentiation and migration (reviewed in [197,198,199]). Importantly, the ECM is dynamically regulated during development as well as under pathological conditions [200,201]. Based on its structural organization and functional complexity, three different forms of brain ECM are identified: the interstitial matrix, the perineuronal nets (PNNs), and the basement membrane (Figure 5) [202]. In this section, we will outline the function of these different forms of ECM in vivo and discuss methods to model them in BoC models.

The brain interstitial matrix constitutes the parenchyma in which brain cells are embedded [202]. It is a complex network that differs considerably in composition from the vascular basement membrane and from systemic ECM outside of the brain [203,204]. Four main components make up the interstitial matrix ECM: hyaluronan (also known as hyaluronic acid), laminin, proteoglycans, and tenascins. Hyaluronan is synthesized and secreted by neurons [205], while proteoglycans are produced by glial cells and neurons [206]. The most common ECM proteoglycans are from the lectican family (versican, aggrecan, neurocan, brevican), which bind to hyaluronic acid [206]. Interstitial ECM also consists of fibronectin, elastin, entactin [207], matrix metalloproteinases (MMPs) capable of remodeling the ECM, [208] and serine proteases [209]. The absence of a collagen component to reinforce this network results in a low stiffness of 1–3kPa [210,211], which is important in glial migration as well as neuronal projections. Stiffness of interstitial ECM has been shown to increase in pathological processes including traumatic brain injury [212] and neurodegenerative diseases [213]. This can profoundly affect the migration of neurons and glia to establish connections and clear waste. 

PNNs are specialized ECM structures that surround neurons [214]. They are composed of hyaluronan backbones that are noncovalently connected to proteoglycans of the lectican family [206]. Aggrecan—a member of the lectican family that is secreted by both neurons and astrocytes—has been shown to play a central role in the formation of PNNs in vivo [215,216]. The functional roles of PNNs in the brain is not fully understood and have been primarily associated with development and maturity of inhibitory neurons [217]. In line with PNN detection in vivo, PNNs have also been observed in a dissociated hippocampal culture maintained for 2–3 weeks in vitro [218], but it is yet to be determined whether mono- or cocultured neurons develop PNNs in vitro. Stiffness of these specialized and localized pockets of ECM is not well-defined.

The cerebrovascular basement membrane (BM) is a specialized ECM secreted from endothelial cells, astrocytes, and pericytes that serves as a barrier between the endothelium and the brain parenchyma. At the level of the capillary, the ECM from each individual cell type is indistinguishable, while at other points along the vasculature (i.e., artery, postcapillary venule), there is more of a separation between endothelial and astrocytic ECM, either by layers of smooth muscle cells, or the perivascular space. Five key proteins make up the cerebrovascular BM: collagen-IV, laminins, nidogens, heparan sulfate proteoglycans (HSPGs), and fibronectin, but there are several other glycoproteins and soluble factors, including growth factors, embedded within (reviewed in [219,220]). Importantly, laminin has three variable chains—making 16 possible isoforms—but only five have been detected in the cerebrovascular BM: laminin-111, -211, -411, -511, and -421 [120,221,222], with different ratios at different points along the cerebrovasculature. Self-assembly of the BM begins with the laminins forming a sheet, followed by the binding of nidogens and HSPGs, and then the binding of a collagen-IV network to the nidogens in order to stabilize the overall structure [223,224]. 

This BM network has been evaluated to have inconsistent elastic moduli values due to the ongoing challenge of obtaining in vivo measurements [225]. Elastic moduli measurements obtained at the capillary level are limited, and at the artery level, the results are variable between species and location of measurement [226,227,228]. Notably, the elastic moduli of arteries will differ from those of capillaries, as collagen-I present in arteries forms thicker, stiffer, and longer fibers than the mesh-like networks of collagen-IV in the microvasculature [229]. Cells adhere to components of the BM via cellular receptors called integrins and dystroglycans, and this adherence plays an integral role in BMECs forming tight junctions [230,231]. Several studies have shown that BM composition is critical in BBB formation, with an emphasis on the importance of both collagen-IV [151,230,232] and astrocytic laminin-211 [107] in the expression of adherens junction marker claudin-5 by BMECs. BM composition is also important in maintaining the integrity and differentiation of mural cells [233], which further ties into BBB integrity given that the differentiation stage of pericytes is critical in their role of maintaining the BBB [64,145]. 

#### 3.3.1. Modeling the Cerebrovascular ECM In Vitro

Traditionally, the ECM component of BBB and NVU in vitro models has solely been used to promote cell adherence, with little consideration of the ECM interactions with cells and their effects on cell phenotypes. Importantly, most of the previously employed coatings and hydrogel systems do not mimic the physiological compositions and functional capabilities of the ECM in vivo [234]. Substrate stiffness dictated by some of these ECM components may also alter the ECMs that the cells in the system produce [235], pushing the model further away from physiological relevance. With the dawn of a new era in ECM research, it has become clear that ECM remodeling is an important process in the context of neurodegenerative disease including Alzheimer’s disease and stroke (reviewed in [219,220]), and therefore it is an increasingly important aspect in the design of in vitro models. The choice of ECM formulation will highly depend on the microsystem under investigation. Substrate stiffness can affect cell phenotypes in vitro. For instance, HUVECs cultured on soft micropatterned polyacrylamide substrates (2.5 kPa compared to 8.5 and 25 kPa) had fewer actin fibers and a rounded nucleus, suggesting that substrate stiffness produces internal tension and remodels the EC nucleus [236]. Bastounis et al. also demonstrated that HUVECs and human microvascular endothelial cells (HMEC-1) had increased traction stresses when cultured on stiff (70 kPa) compared to soft (3 kPa) substrates, but no major changes in their transcriptome were observed, and these changes differed between the cell types [237]. Another study using primary human BMECs found that cells cultured on collagen-coated polyacrylamide gels and collagen-coated glass formed the highest percentage of mature junctions when cultured on gels with a Young’s modulus of 1 kPa, compared to 8 kPa, 15 kPa, 194 kPa, and glass (~47.7 GPa) [12]. In contrast, Katt et al. assessed the effect of matrix stiffness on iPSC-derived BMEC monolayer formation and observed a trend of increasing cell coverage with increasing stiffness of a collagen-I hydrogel (4–7 mg/mL) coated with collagen-IV/fibronectin [35]. Based on the conflicting evidence from different BMEC cell sources to date, we recommend fine-tuning substrate stiffness to be optimized with the selected BMEC to ensure confluent monolayer formation and expression of key markers.

The effects of stiffness are not exclusive to endothelial cells, as a study using primary rat astrocytes demonstrated that a stiffness of 8 kPa induced reactive astrocytes (astrogliosis) in vitro [140], while a lower stiffness of 200 Pa did not. Further studies using primary rat astrocytes demonstrated that astrocytes cultured on substrates with a shear modulus of 10 kPa had increased perimeters, areas, diameters, elongations, and number of extremities compared to substrates with a shear modulus of 100 Pa [238]. Additionally, astrocytes reacting to stiff environments have been demonstrated in Alzheimer’s disease modeling, where the astrocyte mechanosensing ion channel, Piezo1, was upregulated in the presence of amyloid-beta plaques [239]. Georges et al. studied cortical rat astrocyte and neuron morphologies when in contact with soft and hard polyacrylamide substrates. They found that astrocytes had reactive characteristics, such as a spread morphology, when cultured on hard (9 kPa) substrates. However, neurons experienced neurite extensions on both the soft (200 Pa) and hard (9 kPa) substrates [14]. This is in contrast to other studies, which demonstrated dendrite branching increased with substrate stiffness up to 3 kPa [240] and between 100 and 10 kPa compared to soft substrates of ~10 Pa [241]. 

In addition to stiffness, it is important to consider methods to replicate the unique nonlinear rheological properties of the native brain ECM. This increase in stiffness caused by an increase in strain (strain stiffening) is difficult to replicate in vitro as most synthetic materials that are used as a culture substrate for cells do not display these properties [242]. For example, in the case of PET (a common synthetic material that acts as a cellular barrier in 2D BBB BoC devices) the stiffness of the material typically does not change when strain is applied [243]. Undoubtedly, in this case, an ECM coating will be applied to the membrane to enhance cell growth—but since cells are able to sense stiffnesses up to 20 microns deep [244], and the thickness of the vascular BM is 20–200 nm [219], then moving away from membranes with a linear MPa stiffness to soft (kPa range), nonlinear biological materials such as collagen [245] and fibrin [246] would enhance physiological-relevance. When such materials are incorporated into BoC platforms, the shear stress environment should be tailored to trigger this behavior; greater than 0.01 MPa [247].

##### Hydrogels for BoC Modeling

3D cell culture provides an opportunity to mimic a more physiologically relevant cellular environment yet raises challenges in ECM scaffold design to support a complex environment. Both natural and synthetic hydrogel systems have been tested, each with their own unique benefits and limitations. Hydrogels are reticulated structures of crosslinked polymer chains with very high water contents, and they exhibit flexibility in mechanotransduction properties unlike nanofibrous or microporous scaffolds [248]. The field of regenerative medicine continues to benefit from the development of smart hydrogels that are thermo, photo, electro, pH and biochemically responsive [249]. These hydrogels in combination with unique ECM formulations can facilitate development of sophisticated BoC models. Natural hydrogels incorporate ECM components such as collagen, fibrin, hyaluronic acid [250] and are biocompatible due to presence of endogenous factors required for cell viability, proliferation, and development. Another commonly used complex ECM formulation is the commercially available Matrigel^®^, which is a purification of ECM secreted by a murine sarcoma cell line in vitro. While it retains the complexity of endogenous ECM, it is highly variable from lot to lot, contains several growth factors, and is generally poorly defined—making it difficult to precisely identify ECM proportions and their impact on cellular functions. 

For many years, gelatin has been used as a cell culture substrate for in vitro and in vivo applications due to its advantageous traits of being readily available, biocompatible and biodegradable [251]. Gelatin is a natural, hydrophilic polymer that is produced from denatured collagen and possesses arginine-glycine-aspartic acid peptides that encourage cell adherence, proliferation and differentiation [252]. This was demonstrated when iPSC-derived BMECs were successfully seeded into the lumen of a 3D gelatin structure to form a stable monolayer [44]. However, gelatin’s need for chemical crosslinking can sometimes lead to local cytotoxicity, and its poor thermal stability at body temperature [253] led to the widespread use of methacrylamide-modified gelatin (GelMA) [254]. GelMA’s ability to be mechanically tuned by adjusting its polymer [255] concentration, initiator concentration and ultra-violet (UV) or visible light conditions has enabled its use in biomedical applications [254]. For example, GelMA was used as a treatment for rats following myocardial infarction [256]. GelMA’s ability to be mechanically tuned between 1–200 kPa [257] has enabled the identification of favourable conditions for adherence of HUVEC [258] and PC12 cells [259]. Furthermore, GelMA has been further biofunctionalized with an N-cadherin extracellular peptide that has been shown to enable iPSC-derived neurons to form networks with functional synapses [54]. 

Hyaluronic acid is a major component of the interstitial ECM, and its biocompatibility, biodegradability, and ability to be crosslinked render it a prime candidate for in vitro hydrogel-based studies [260]. Methacrylated hyaluronic acid (Me-HA) enabled iPSC-derived neural progenitor cells (NPCs) to be cultured within hydrogels with stiffness values of 0.5 and 2 kPa [255]. A study that added gelatin and gelatin/HA into rats demonstrated that gelatin/HA had better contiguity with surrounding tissues [261]. 

Fibrin is often used in in vitro neuron-associated studies due to its presence in peripheral nerve-repair [262] and its ability to be mechanically adjusted based on the concentrations of thrombin and fibrinogen [262]. Fibrin has been able to achieve a stiffness range of ~2–85 kPa [241]. Although fibrin is not present in the interstitial ECM, fibrin gels have been used in several in vitro models that leveraged HUVECs and iPSC-derived BMEC as their endothelial cell sources [263,264,265]. However, recent work has shown that fibrin is easily digested by iPSC-derived BMEC and is therefore not ideal to use with this cell type [35]. 

Collagen-I is a very popular hydrogel used in many models of the microvasculature [42,47,50], including models adopted for the commercially available Mimetas Organoplate^®^ platform [39]. Rat-tail collagen-I can be mechanically tuned by adjusting the collagen concentration, the gelation temperature and the solubilization technique. Collagen-I at a concentration of 4 mg/mL was demonstrated to have a storage modulus—the materials’ ability to store energy elastically [266]—of ~100 Pa, and when 10 mM of genipin was added, this increased the modulus to ~300 Pa before experiencing nonlinear stiffening up to ~700 Pa [245]. 

Many of the abovementioned natural hydrogels have been optimized or combined to be able to form a perfusable 3D BoC and be compatible with cell types found in the BBB (see Table 1). Further optimization of such gels could be beneficial in the development of models. For instance, exogenous addition of bovine aggrecan in hydrogels for cartilage regeneration has shown promising outcomes but these aggrecan formulations have yet to be tested in CNS models [267]. 

##### Coatings to Mimic Cerebrovascular Basement Membrane In Vitro

Most cells in culture secrete their own ECM, which promotes their attachment to the culture surface. Thomsen et al. recently demonstrated that in a Transwell system, primary murine BMECs deposit a basement membrane that closely resembles the composition seen in vivo [222], although the cells were cultured onto a membrane that was precoated with collagen-IV and fibronectin. This is common practice in BBB/NVU modeling since an ECM substrate facilitates cell adhesion, promotes BMEC monolayer formation, and induces tight/adherens junction expression to some degree; all of which ultimately dictate downstream measures including TEER and permeability assays. Several groups have investigated the effect of different coating compositions on the phenotype of immortalized, primary, or iPSC-derived human BMECs, with results that seem to depend on the type of cells used. It is clear that iPSC-derived cells show the most varied responses to differences in coatings, while immortalized cells do not show much of a response to any coating, and primary cells responses vary. Here, we will provide an overview of the selection of compatible BM coatings for each source of endothelial cells as they are the cell type most affected by BM composition. Out of all endothelial cell options, immortalized cell lines are the most readily cultured, and many studies do not use an ECM coating at all [270,271]. Since HCMEC/D3 cells are the most commonly used BMEC cell line, we will focus on coatings compatible with these cells. Given their generally robust phenotype (reviewed in [79]), most studies that do use a coating for these cells select generic rat-tail collagen-I (10 ug/cm^2^) [272,273,274,275] due to its low cost, even though collagen-I is not found in the cerebral microvasculature. Several combinations of coatings that are more physiologically relevant have been tested in an attempt to optimize the barrier formation of HCMEC/D3, with little success. In a recent study by Hinkel et al. using Transwell inserts, combinations of coatings with collagen-I, collagen-IV, fibronectin, and laminin were tested, but the condition without any additional coating on the PET insert gave the highest mean TEER value (17.7 Ω·cm^2^) [80]. Further research is warranted to investigate coatings as a variable that may influence HCMEC/D3 barrier formation, but to date there is no evidence that immortalized BMEC phenotypes change in response to increasing complexity of BM coatings. Based on these results, HCMEC/D3 can be cultured without a coating to conserve costs; however, this should be carried out only after considering the constraints and limitations that result when choosing immortalized cell lines and/or a nonphysiological microvascular BM substitute.

To our knowledge, there is no consensus on the optimal ECM for use with primary BMECs. In a 2013 study, primary HBMECs in monoculture did not have a significantly higher TEER when cultured on a collagen-IV/fibronectin (80 ug/cm^2^, 20 ug/cm^2^) or Matrigel^®^ (80 ug/cm^2^) coating compared to collagen-I (10 ug/cm^2^) [75]. In the past year, a more comprehensive analysis on culture conditions of HBMEC has been carried out by Gray et al. who explored stiffness, coatings, and additional media supplements to optimize mature tight junction expression. They tested a series of coatings, including collagen-I (100 ug/mL), fibronectin (100 ug/mL), collagen-IV (100 ug/mL), laminin (2 ug/cm^2^), and 0.4% thiol-modified hyaluron: 0.4% thiol-modified gelatin, with some combinations of the mentioned coatings, and they quantified tight junction phenotype (continuous, punctate, or perpendicular) [12]. They found that the fibronectin coating marginally induced the greatest mature tight junction coverage compared to the other coatings, which is aligned with previous studies using porcine BMECs that demonstrated the importance of fibronectin, collagen-IV and laminin for in vitro barrier formation [231,276]. Many groups use a generic collagen-I coating to culture primary BMECs, but a recent study observed dedifferentiation of primary murine BMECs cultured on collagen-I-coated plates [83], indicating that collagen-I alone is not sufficient to maintain the phenotype of BMECs in the absence of other stimuli. To our knowledge, no study has fine-tuned ECM composition to culture BMEC with other cell types. 

Despite emerging questions surrounding their true identity [92], iPSC-derived brain microvascular endothelial cells remain the only BMEC capable of barrier formation reaching physiological levels seen in vivo. A substrate mimicking the BM is critical for their adherence and complete differentiation, with the majority of existing protocols opting for a collagen-IV (400 ug/mL) and fibronectin (100 ug/mL) mixture as the final “purification” step in the differentiation [26,44,87,88,89,90,91]. Importantly for 2.5D and 3D applications, collagen-IV/fibronectin coatings increased TEER of iPSC-derived BMEC on a collagen-I hydrogel in a Transwell insert [35], and another group demonstrated that an iPSC-derived BMEC monolayer demonstrated a stable barrier for up to 21 days when cultured in a gelatin channel coated with collagen-IV/fibronectin [44]. Given this evidence, collagen-IV/fibronectin coatings appear to be suitable for iPSC-derived BMEC culture. 

The other most commonly used substrate for iPSC-derived BMECs is commercially available Matrigel^®^. While Matrigel^®^ is an attractive option given its complexity and compositional alignment with many of the constituents of the cerebrovascular BM (collagen-IV, laminins), it should be noted that it only contains laminin-111 [277], and therefore lacks laminin-211, which has been shown to be key in the context of BBB integrity [107]. A study by Sixt et al. demonstrated that laminin-111 is predominantly synthesized by leptomeningeal cells in large vessels, and it is either absent or produced at low levels at the level of the capillary [278]; thus coatings that are predominantly laminin-111 are not the optimal option to recapitulate the capillary microenvironment in vivo. The recognized lot-to-lot variability in Matrigel^®^ composition has led researchers to examine specific components of ECM to generate a more controlled and reproducible environment with less lot-to-lot variation. A recent study compared defined coatings to Matrigel^®^ for the differentiation of iPSC-derived BMECs [279] and found that cells cultured on laminin-221 had superior barriers, with higher TEER values, and the barrier was maintained for longer. Though laminin-221 is slightly different from the laminin-211 secreted by astrocytes, they both share the alpha-2 chain, which is suggested to be the main regulator of the maturation and function of the BBB [107]. 

#### 3.3.2. ECM Choice Special Considerations

Adult vs. developmental ECM formulations: Physiologically, the composition of the ECM differs between the developing and the adult brain, where the former is much more plastic than the latter. ECM components are also differentially secreted, and some are only expressed in the developing brain. Therefore, it is important to consider the age and maturation parameters of the cells being cultured to better identify the appropriate ECM formulation.

Downstream Quantification of ECM: An important consideration when downstream analysis will involve ECM quantification is how to distinguish endogenous ECM from the coating. One method is to use ECM coatings from a different species than the cells populating the model. Thomsen et al. took this approach and used human collagen-IV/fibronectin to coat the Transwell membranes of their primary murine cell model [222]. This enabled them to identify human sequences during mass spectrometry analysis of the deposited ECM. Another approach is to quantify mRNA from harvested cells, but mRNA is not always reflective of protein production or deposition [280]. Additional approaches to take include using collagen-I or GelMA as the ECM, since collagen-I is not produced in the microvasculature, and gelatin is mainly derived from sources that are rich in collagen-I [281].

Drug Delivery: In vitro assessment of BBB permeability a priori requires a tight and physiologically relevant barrier. This will involve choosing a BMEC source that is capable of forming continuous tight junctions and providing a microenvironment to preserve its phenotype. The barrier produced by the vascular basement membrane must also be considered, in addition to the endothelial monolayer itself. Here, complexity of the ECM coating is critical. One group has shown that using laminin and/or collagen-IV on their own was not sufficient in providing the filtering effect—suppressing microscopic mobility—that endogenous ECM displays [282].

### 3.4. Microenvironment Criteria

**Figure 6:**
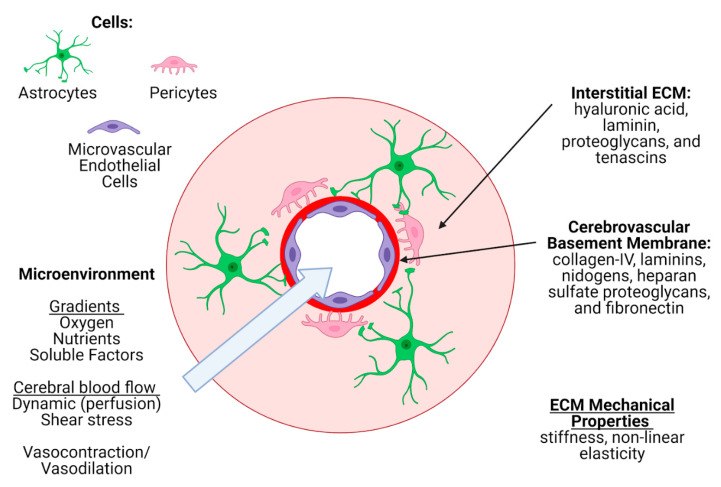
Components included in an MVM of the BBB.

The brain is a complex system with cells and ECMs representing only a portion of the brain microenvironment. Once appropriate cells and ECMs have been selected for the MVM the next step is to determine additional components that may need to be present in order to obtain a functional model. These components include nutrient and oxygen supply, waste removal and their respective gradients (Figure 6).

In vivo, brain homeostasis is a complex phenomenon that has many contributing factors. For instance, there are natural concentration gradients for oxygen, pH, nutrients and cellular metabolites that affect various cell behaviors (i.e., cell signaling) [55]. These gradients are highly dynamic and are driven by blood flow, as well as the movement of two other key fluids: the cerebrospinal fluid (CSF) encasing the entire central nervous system, and the interstitial fluid (ISF) between cells in the brain parenchyma.

In vivo, cerebral blood vessels supply oxygen and nutrients to the brain in response to the metabolic activity of neurons through neurovascular coupling [283,284]. Neurovascular coupling involves the cued vasodilation of the microvasculature by the neurons. Vasodilation and vasoconstriction are the main mechanisms that control oxygen and nutrient levels, with recent evidence showing that pericytes play a crucial role in regulating the vasodilation in the microvasculature [285]. However, this dynamic aspect of the brain microenvironment is often overlooked, thus limiting physiological relevance and predictability. Replication of this aspect of the brain microenvironment involves incorporation of the various NVU cells as well as the selection of an appropriate ECM which should be soft to enable the expansion and contraction in response to mural cell activity. Replication of this cell–ECM interaction can be assisted by selecting an ECM substitute that can be remodeled by secreted MMPs, and displays nonlinear elasticity. 

The NVU utilizes passive diffusion as well as selective and active transport to provide cells with the molecules (O_2_), nutrients, ions and macromolecules (i.e., glucose) essential for neural function [286]. Replicating the transport of these components across the microvasculature is important and can be accomplished through microfluidic platforms. The flow within these platforms is laminar (diffusion limited) and often controlled by pump-based systems mimicking the passive diffusion of hydrophobic molecules across the brain endothelium [287]. These systems also provide the opportunity to replicate the microvascular wall shear stress present in the brain vasculature. The shear stress experienced in vivo is known to increase endothelial gene expression and barrier function [9,288]; therefore, replication is important to create a BBB that restricts the diffusion of large hydrophilic molecules and solutes in the circulating blood from nonselectively crossing into the cerebrospinal fluid.

CSF is a specialized filtrate of blood with a stable composition that plays roles in nutrient delivery and waste clearance [289]. It is mainly produced by a structure called the choroid plexus, which is situated within the ventricles of the brain. The dynamics of CSF secretion, flow, and drainage are not well-defined [290] but are a current research interest. Large-scale in vitro models of CSF flow are being developed to understand the flow dynamics and their relationship with pressure in the brain [291], but this complex movement is not able to be captured by current microfluidic models. While the BBB is a main focus of BoC modeling, the blood–CSF barrier produced by choroid plexus ependymal cells is also an important site for studying many processes, including immune responses in the context of the brain [292]. A review by Erb et al. outlining efforts to model the blood–CSF barrier was recently published [293].

ISF is also a critical component of the microenvironment in the brain, which feeds into the CSF since there is no distinct barrier between the two fluids. Waste produced by the cells in the brain can diffuse from the ISF to the CSF for clearance or can follow perivascular clearance pathways along the basement membrane of cerebral vessels to ultimately be cleared out through the leptomeningeal arteries on the surface of the brain. Perivascular clearance pathways were first discovered in mice intracranially injected with fluorescent dyes [294] and have since gained considerable attention in neurodegenerative disease research, where these pathways seem to get disrupted [294]. This pathway is thought to be driven by arterial pulsations [295,296], which are a challenge to recapitulate with microfluidic models. A review on the interstitial system of the brain along with limitations in its modeling in vitro was recently published by Lei et al. [297]. They emphasize that the ECM and geometry of the model will influence the trajectory of metabolite transport, further reinforcing the development of 2.5/3D models with physiological ECM.

Establishing these complex environments requires consideration of the choice of ECM as well as the design of the chip. For example, the presence of blood vessels influences natural gradients; therefore, it is important to consider the distance from the blood vessel to the region of interest within the in vitro model. This can be accomplished by designing the device to contain microchannels separated by thin membranes [269], hydrogels with embedded vascular structures [298] or using self-assembly based techniques [265]. In addition, the diffusion of molecules through the ECM also impacts cellular metabolism and the production of waste products—further highlighting the importance of selecting an appropriate ECM. Traditional 2D monolayer cultures are not suitable for establishing oxygen and nutrient gradients as all cells are homogeneously exposed to the tissue culture media. In order to establish physiologically relevant gradients, cellular cultures encapsulated in 3D matrices and/or microfluidic platforms are needed [55]. Additionally, replication of brain homeostasis can be optimized using growth factors and additional proteins in the media or hydrogel environment. Astrocyte conditioned media containing cyclic adenosine monophosphate (cAMP) or the addition of Rho-associated kinase (ROCK) inhibitor combined with cAMP has been shown to facilitate a more BBB-like monolayer in HUVECs [299], primary HBMECs [34] and iPSC-derived BMECs [35].

## 4. Discussion

**Figure 7:**
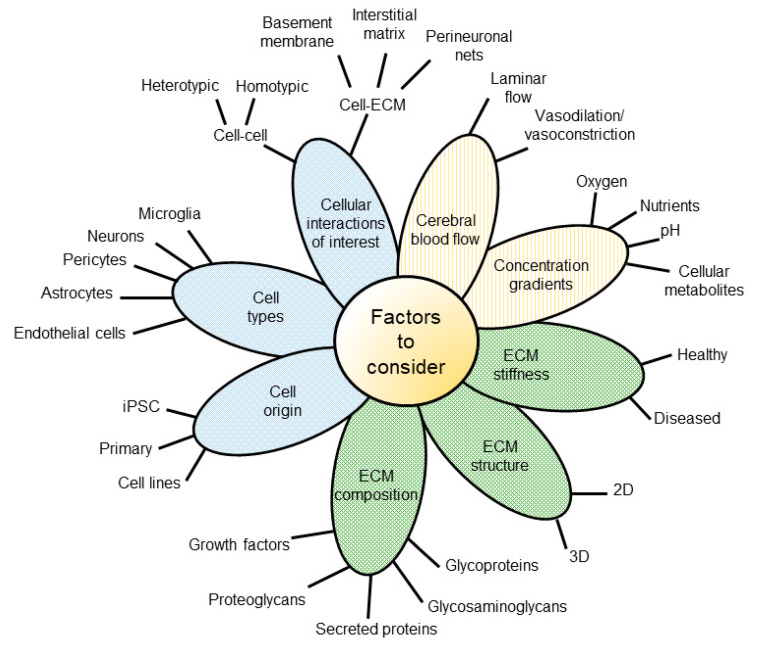
Factors to consider when designing BoC models.

Here, we describe the factors to consider when designing and selecting fit-for-purpose BoC models of the BBB and NVU (Figure 7).

When modeling the NVU it is essential that the MVM incorporates brain microvascular endothelial cells, pericytes, astrocytes, and neurons. All four cell types are needed to replicate the cell–cell interactions necessary to interrogate the biology related to the NVU. Inability to incorporate these cell types may result in an oversimplified model that yields misleading results. However, work focused on interactions and functions of the BBB can benefit from a simplified MVM by using BMEC, astrocytes and pericytes to assess cell physiology and barrier function. 2D BBB BoC models can be utilized for simple biological questions. For instance, Park et al. developed a 2D BBB BoC model that was used to study the effects of differentiation of iPSC-derived BMECs under hypoxic conditions. Their results show that hypoxic conditions coupled with shear stress exerted on the endothelium results in maximum TEER values of 24,000 Ω [26], suggesting that this protocol could be a useful resource for BoC developments. However, the inherent nonphysiological stiffness and planar geometry of 2D BoC models will automatically limit astrocyte projections that are observed in cultures grown in a 3D matrix; therefore, utilizing microfabrication techniques to develop 2.5D and 3D BoC models that incorporate a hydrogel as the ECM is advisable. To ensure BBB and NVU models are not being overestimated on their physiological relevance, more emphasis should be placed on the reasoning behind why certain models are being utilized over others when studies are being reviewed and published. This includes investigating the effects of substrate material and stiffness on cell types independent of shear and media composition before moving to co-, tri- and quadculture models. Although it is important to increase physiological relevance through increased complexity to obtain more predictive results, it is also essential to limit the number of dependent variables that a model has so that the results can be easily interpreted. Increasing complexity in a controlled manner—one dependent variable at a time—will facilitate the ease of interpretation as well as troubleshooting when the model behaves in a nonphysiological manner. 

The suitability of a model will depend on multiple variables including the question of interest, application, resource availability, cost and throughput capacity. However, once selected, it is important to understand the limitations of the device prior to using it as an experimental tool. For example, the benefits and limitations of immortalized cell lines, primary cells and iPSC-derived cells should be considered before use in vitro. While iPSC-derived cells are currently seen as the most attractive option, there is considerable room for improvement in the quality, purity and maturity of differentiated cells, as reviewed elsewhere [179,189,300,301]. Depending on the origin of the cell types of interest, the composition of ECM incorporated into the models should also be considered. BM coating considerations for endothelial cell phenotypes should be cell type-dependent, as immortalized cell lines have similar phenotypes across different ECM substrates or without coatings [270,271,272,273,274,275], primary cells have shown promising results with fibronectin, collagen IV and laminin as the BM [231,276] and iPSC-derived BMECs show physiological behavior when cultured with collagen IV/fibronectin coatings, Matrigel^®^, and possibly laminin-221 [44,279]. Any investigators studying barrier function should consider testing an array of BM compositions to find a suitable option for their specific cell source. To determine suitability, quantitative analysis methods should be used, such as confluency percent, tight junction coverage and tight junction maturity [12,34,35]. Further, there is much room for improvement of mechanically stable hydrogels that can encompass brain cells while also forming a stable lumen. Available options that have been used with primary astrocytes and pericytes include collagen-I, fibrinogen, and hydrogel combinations that include hyaluronic-acid and Matrigel^®^ [43,45,50] When selecting an ECM, it is important to optimize the stiffness to induce healthy cell phenotypes, as BMEC, astrocytes, and neurons are known to respond differently to different substrate stiffnesses [14]. Additionally, the method of quantifying the stiffness of hydrogels should be considered as there are different forms of elastic moduli (compressive, tensile) and depending on the instrument (dynamic mechanical analyzer, tensile testing machine) and the conditions of the sample during measurement (hydrated, nonhydrated), conflicting elastic modulus values may be obtained. 2.5D and 3D BoC models should encompass an optimized microenvironment that aims to nourish the cells while providing physiological flow to induce BMEC gene expression [9,288].

As these BoCs are intended to be used as in vitro tools, it is not only essential that the MVM replicates the microarchitecture and microenvironment of the brain but also can be created in a reproducible manner. The lack of standardization of organ-on-a-chip devices has been identified as a major barrier for adoption by the broad research community. Therefore, it is important that once designed, the MVM be validated for reproducibility and robustness. A recently developed open-source microphysiology system database aims to provide a centralized data center to allow for investigators to evaluate the reproducibility of their model and compare it with clinical data [302]. Once validated in-house, it is also necessary to have the MVM tested and validated by an external party to remove bias from the process. This was demonstrated by Sakolish et al. who used predefined metrics and an external laboratory to determine the robustness and reliability of a human microfluidic four-cell liver acinus microphysiology system (LAMPS) [303]. Tissue chip testing centers have been set up to evaluate functionality, reproducibility, robustness and reliability of chips as their primary initiatives [304]. 

## 5. Conclusions

Deciding on factors to include in a microfluidic BoC platform can be a complex decision-making process that includes many external factors. In this review, we have highlighted that the presence of BM, ECM, stiffness, flow, nutrient, and growth factors influence cell physiology and promote a physiological model of the microvasculature. We have also provided an overview of 2D, 2.5D and 3D BoC models that have been successfully used. Future developments of BBB or NVU models should prioritize building in physiological relevance into their models to ensure that cells are behaving as they would in vivo. This review has focused on the design from the perspective of the physiological model at the innovation and research stage. 

Finally, BoC systems offer the opportunity for better in vitro methods within the drug development process. Within this workflow, 2D monoculture high throughput assays may be used during initial screening of upwards of 10,000 compounds. As candidates move through the pipeline, these BoC systems may be positioned in the preclinical trials, enabling teams to gain more predictive data prior to moving to animal testing of a narrowly focused number of drug candidates. BoC systems can allow researchers to examine some of the mismatches between the hits from 2D screens and the failure of those drug candidates in animals, and testing a well-defined panel of drug candidates can help elucidate the biology. Pharmaceutical companies may also need integrated platforms that feature multiple-organ models. In future, integrating organ-on-a-chip devices into automated equipment will greatly improve their usability, lowering the barrier to wider adoption.

As the BoC models move beyond prototyping in a laboratory setting toward routine adoption in the industrial setting, users will need to consider alternative microfabrication methods more suited for high volume production. Standards, guidelines, and harmonization of materials, standardization of interconnects between modules to permit plug-and-play functionality, and standardized common testing methods will not only help the researchers to develop new systems faster and with higher success, but will also facilitate translation from research labs to industry. 

## Figures and Tables

**Table 1:** Hydrogels and coatings previously used in 2D, 2.5D and 3D BoC devices.

BoC Dimension	ECM Hydrogel	BM Coating	Endothelial Cell Type	Coculture Cell Types	Tracer and Barrier Permeability	TEER (Ohm/cm^2^)	Ref
2D	N/A	collagen IV and fibronectin	iPSC-derived BMEC	primary astrocytes, pericytes and EZ spheres differentiated into astrocytes and neurons	3 kDa Dextran: 1 × 10 ^−7^ cm/s	1500	[268]
2D	N/A	collagen IV and fibronectin	iPSC-derived BMEC	primary human pericytes and astrocytes	3, 10, 70 kDa Dextran: 8.9, 1.1 and 0.24 × 10^−8^ cm/s, respectively	24,000	[26]
2.5D	collagen I	collagen I	hCMEC/D3 and HUVEC	primary rat astrocytes and neurons	10 kDa Dextran: 1.23 × 10^−5^ cm/s	N/A	[269]
2.5D	collagen I	N/A	Primary HBMEC	primary human pericytes and astrocytes	3 kDa Dextran: 2–3 × 10^−6^ cm/s	N/A	[42]
3D	collagen I, Matrigel^®^, hyaluronic acid (HA)	N/A	hCMEC/D3	human astrocytes	4 Da FITC dextran: 0.7 × 10^−6^ cm/s	~1000	[43]
3D	porcine gelatin	collagen IV and fibronectin	iPSC-derived BMEC, HUVEC, human dermal microvascular endothelial cells (uVas)	N/A	3 kDa Dextran: 2.9 × 10^−7^ cm/s	N/A	[44]
3D	collagen I crosslinked with genipin	collagen IV and fibronectin	iPSC-derived BMECs	N/A	Lucifer Yellow: 5–6 × 10 ^−7^, Alexa 647: below detection limit and 10 kDa Dextran: Below detection limit	200–4000	[50]
3D	collagen I	collagen IV and fibronectin	iPSC-derived endothelial cells	hiPSC pericytes	Lucifer Yellow: 4 × 10^−7^ and 10 kDa Dextran was below detection limit	N/A	[37]
3D	fibrinogen	N/A	HUVECs	human brain pericytes	Dextran Rhodamine B: 70 kDa: 2.62 × 10^−7^ cm/s	N/A	[45]
3D	collagen I, HA, Matrigel^®^	N/A	hCMEC/D3	primary human astrocytes and human coronary arterial smooth muscle cells (HCASMCs)	4 kDa FITC dextran: ~1.5 × 10^−6^ cm/s	N/A	[46]

## Data Availability

Not applicable.
